# Molecular characterization and phylogenetic analysis of transmissible gastroenteritis virus HX strain isolated from China

**DOI:** 10.1186/s12917-015-0387-8

**Published:** 2015-03-21

**Authors:** Xiaoliang Hu, Nannan Li, Zhige Tian, Xin Yin, Liandong Qu, Juanjuan Qu

**Affiliations:** College of Resources and Environment, Northeast Agricultural University, Harbin, 150030 People’s Republic of China; College of Life Sciences, Northeast Agricultural University, Harbin, Harbin, 150030 People’s Republic of China; College of Wildlife Resources, Northeast Forestry University, Harbin, 150040 People’s Republic of China; National Key Laboratory of Veterinary Biotechnology, Harbin Veterinary Research Institute, Chinese Academy of Agricultural Sciences, Harbin, 150001 People’s Republic of China

**Keywords:** Porcine transmissible gastroenteritis virus, Sequence alignment, Phylogenetic analysis

## Abstract

**Background:**

Porcine transmissible gastroenteritis virus (TGEV) is the major etiological agent of viral enteritis and severe diarrhea in suckling piglets. In China, TGEV has caused great economic losses, but its role in epidemic diarrhea is unclear. This study aims to reveal the etiological role of TGEV in piglet diarrhea via molecular characterization and phylogenetic analysis.

**Results:**

A TGEV-HX strain was isolated from China, and its complete genome was amplified, cloned, and sequenced. Sequence analysis indicated that it was conserved in the 5′ and 3′-non-translated regions, and there were no insertions or deletions in nonstructural genes, such as ORF1a, ORF1b, ORF3a, ORF3b, and ORF7, as well as in genes encoding structural proteins, such as the envelope (E), membrane (M), and nucleoprotein (N) proteins. Furthermore, the phylogenetic analysis indicated that the TGEV-HX strain was more similar to the TGEV Purdue cluster than to the Miller cluster.

**Conclusions:**

The present study described the isolation and genetic characterization of a TGEV-HX strain. The detailed analysis of the genetic variation of TGEVs in China provides essential information for further understanding the evolution of TGEVs.

## Background

Transmissible gastroenteritis virus (TGEV) is the etiological agent of transmissible gastroenteritis (TGE), and it can cause viral enteritis and severe diarrhea with high morbidity in pigs of all ages, as well as high mortality in suckling piglets [1]. It occurs at swine-raising farms and results in significant economic losses [2,3].

TGEV is an enveloped virus belonging to the *Coronaviridae* (CoV) family and the *Nidovirales* order. It possesses a large 28.5-kb single-stranded, positive-sense RNA genome. About two-thirds of the entire RNA comprises open reading frames (ORFs) 1a and 1b, encoding RNA replicase. The 3′ one-third of the genome comprises genes encoding structural and non-structural proteins [4,5]. The genes of TGEV are arranged in the order of 5′-rep-S-3a-3b-E-M-N-ORF7-3′.

The spike (S) gene of TGEV encodes an approximately 1,450-amino acid protein, with a molecular weight ranging from 128–160 kDa without glycosylation and 150–200 kDa after glycosylation. Functionally, the S glycoprotein is the major target of neutralizing antibodies, and it is also related to host cell tropism [6], interaction with its cellular receptor, pathogenicity, fusion, and hemagglutination activity [7-9]. ORF7 encodes a small hydrophobic protein (HP) during viral replication. The intracellular localization of the HP suggests that it may play an important role in the process of membrane integrity during viral replication and/or virion assembly [10,11].

In this report, we isolated a TGEV-HX strain of TGEV from the feces of piglets in Heilongjiang province in China. To better understand the molecular characteristics of this isolate, its complete genome sequence was obtained, and a phylogenetic tree was constructed based on the complete ORF sequence of the S gene. The results provide molecular and phylogenetic information for a Chinese isolate of TGEV, which may assist in elucidating the genetic evolution of TGEV in China.

## Methods

### Ethics statement

Pigs used in this study were approved by the Institutional Animal Care and Use Committee (IACUC) of the Harbin Veterinary Research Institute (HVRI), the Chinese Academy of Agricultural Sciences. No animals were sacrificed specifically for this study. Feces samples were collected at the farm.

### Viral isolation and identification

Five fecal samples were collected from piglets with diarrhea in a suburb of Harbin, the capital of Heilongjiang province, P. R. China. As both TGEV and PEDV can cause diarrhea, two pairs of specific primers (TGEV-NF; TGEV-NR; PEDV-NF, PEDV-NR) were employed to identify the kinds of viruses in the above samples (Table 1). PCRs were conducted as below, and the cycling parameters for the PCR included 94°C for 5 min, followed by 30 cycles of 94°C for 0.5 min, 55°C for 0.5 min, and 72°C for 1 min, and a final extension at 72°C for 10 min. Then, PCR-positive viral samples were inoculated into PK-15 cells, which were grown as a monolayer in Dulbecco’s modified Eagle medium (DMEM) (GIBCO, Grand Island, NY, USA) containing 10% fetal calf serum (GIBCO, Grand Island, NY, USA) and 5% CO_2_ in air. Viruses were passaged three times and were harvested by three cycles of freezing and thawing. Cellular debris was removed by low speed centrifugation at 3,000 × g (Eppendorf, Hamburg, Germany) for 10 min, and the supernatant was aliquoted and stored at −80°C. Viral titers were determined using the Reed-Muench method [12].

**Table 1:** **Primers used for identifying and completely sequencing the TGEV-HX strain**

**Primer**	**Sequence 5′-3′**	**Position**
TGEV-NF	TCATGCAGATGCCAAATTTAAAGA	27,213-27,236
TGEV-NR	TCATCCTTCTTGTTATTGAATTGT	27,456-27,479
PEDV-NF	TTTCTAAGGTACTTGCAAATAATG	26,382-26,405
PEDV-NR	TTGGAGATCTGGACCTGTTGTTGC	26,757-26,780
P1	ATGAGTTCCAAACAATTCAAGA	315-336
P2	ATCAAA ACATCCAAAGCACCCT	4,318-4,339
P3	AATTCA AAGTCCTAA AAACGAT	4,250-4,271
P4	GCGTAGATGATCATA AAGAACG	8,470-8,491
P5	CATTGTCACCCTTGTTGTGAAC	8,420-8,441
P6	GTAGATGTCAAA AGCTCTACTA	12,400-12,421
P7	TCTATGCAGAGTTTTACTGTTG	12,300-12,321
P8	TAATGAATTTATGCTTTGTTCC	15,200-15,221
P9	AGGCATGTGTGTAGTATGTGGT	15,100-15,121
P10	AAGCTTAGCAAA AGCTCTT	18,169-18,187
P11	CGCACTCGCTCTAAATTGTCTT	18,080-18,101
P12	ACTACGTTTAACCGTTGTCTGT	20,660-20,681
P13	ATGAAA AAACTATTTGTGG	20,365-20,383
P14	TTA ATGGACGTGCACTTTT	24,690-24,708
P15	GCTGTGGATGCATAGGTTGTTT	24,608-24,629
P16	TTGGAGGGTTATGGGGTTGAAG	27,021-27,042
P17	CTTCTAAATGGCCAACCAGGGAC	26,910-26,932
P18	CGAGCATCTCGTTTAGTTCGTT	28,056-28,077
P19	AGA AAGGTCAGAGCAAGATGTG	27,963-27,984
P20	GTATCACTATCA AAAGGA AAAT	28,559-28,580
P-F	CAAACTGAATGGAAATAATCAA	139-160
P-R	ATTTGGCAATGCTAGATTTAGTAA	28,441-28,464

### Extraction of viral RNA, reverse transcribed-polymerase chain reaction (RT-PCR) and complete genome sequencing

Viral RNA was extracted from PK-15 cells infected with TGEV-HX using the TRIzol reagent (Invitrogen, Carlsbad, CA, USA). cDNA was generated by adding 4 μl of RNA to the following components: 4 μl of 5× reverse transcription buffer, 4 μl of dNTPs mixture (2.5 mM), 0.5 μl of RNase inhibitor, 5 μl of random primer (50 μM), 0.5 μl (10 U) of AMV reverse transcriptase, and 2 μl of sterile water. The components were gently mixed in an Eppendorf tube and incubated at room temperature for 10 min, then transferred to a water incubator at 42°C for 1 h prior to storage at −20°C . The resulting cDNA was amplified by PCR using LA Taq DNA polymerase (TaKaRa, Tokyo, Japan). Ten pairs of primers were designed based on conserved regions of TGEV strain H165 (Table 1). PCRs were conducted in a total of volume of 50 μl containing 5 μl of 10× buffer, 3 μl of dNTPs mixture (2.5 mM), 8 μl of cDNA, 1 μl of forward primer (10 μM), 1 μl of reverse primer (10 μM), 5 U of LA Taq polymerase (TaKaRa, Tokyo, Japan), and 31 μl of sterile water. The cycling parameters for the PCR included 94°C for 1 min, followed by 30 cycles of 94°C for 1 min, 50°C for 1 min and 72°C for 1 min, and a final extension at 72°C for 10 min. Two primers (P-F, P-R) were employed to confirm the 5′ and 3′ ends of the viral genome by rapid amplification of cDNA ends (RACE) using the RACE cDNA Amplification Kit (Invitrogen, Carlsbad, CA, USA). The PCR products were run on agarose gels, and correctly sized amplicons were observed. Then, the PCR products were purified using the Axygen Gel Extraction Kit (Axygen, USA) and cloned into pMD18-T (TaKaRa, Tokyo, Japan). Three to five independent clones of each TGEV amplicon were sequenced. The DNA was sequenced using an ABI 3730XL Sanger-based Genetic Analyzer (Applied Biosystems, Waltham, MA, USDA).

### Electron microscopy

PK-15 cells infected with TGEV were harvested by freezing and thawing three times. One mL of cell culture was centrifuged for 5 min at 800 × g. The supernatant was transferred into a new microfuge tube and centrifuged for 10 min at 13,400 × g. Then, the pellet was negatively stained with 2% phosphotungstic acid and analyzed on a transmission electron microscope (H-7650, Hitachi, Tokyo, Japan) [13].

### Sequence alignment and phylogenetic analysis

Sequence data were assembled and analyzed using Clustal X software (1.83) and DNASTAR. To determine the relationship between the TGEV representative isolates and the HX strain, phylogenetic trees based on the S gene were constructed using molecular evolutionary genetics analysis (MEGA) software (version 4.0) using the neighbor-joining (NJ) method. Bootstrap values were estimated for 1,000 replicates. The sequences of the TGEV reference strains were obtained from GenBank, and the details are summarized in Table 2. The sequences obtained in this study were assembled and submitted to GenBank under the accession number KC962433.

**Table 2:** **Source of transmissible gastroenteritis virus (TGEV) sequences used in the experiment**

**Isolate**	**Accession no.**	**Origin**	**Collection date**
96-1933	AF104420	UK	2001
H16	FJ755618.2	CHN	2010
H165	EU074218	CHN	2010
TGEV-HX	KC962433	CHN	2013
ISU-1	DQ811787	USA	2009
Miller 6	DQ811785	USA	2009
Miller 60	DQ811786.2	USA	2011
Purdue	DQ811789	USA	2011
Purdue 115	DQ811788.1	USA	2009
SC-Y	DQ443743	CHN	2006
TS	DQ201447	CHN	2006
WH-1	HQ462571.1	CHN	2011

## Results

### Virus culture and electron microscopy analysis

**Figure 1:**
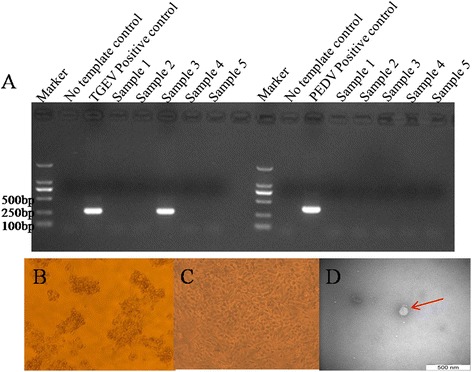
**Identification and Isolation of TGEV-HX. (A)** Identification of five transmissible gastroenteritis virus (TGEV) samples by PCR.** (B)** Cytopathic effect (CPE) induced by TGEV HX in the PK-15 cell line. **(C)** control (uninfected) PK-15 cells. **(D)** Electron micrograph of the purified isolate negatively stained with 2% phosphotungstic acid. The scale bar represents 500 nm.

The PCR results confirmed that one of five samples was TGEV-positive, designated TGEV-HX; the other four samples were TGEV-negative and all five samples were PEDV-negative (Figure 1A). After three passages, cytopathic effects (CPE) were found in the PK-15 cells, as evidenced by the cells rounding up and enlarging, the formation of syncytia, and the detachment of cells into the medium (Figure 1B and C). The median tissue culture infective dose (TCID_50_) of TGEV-HX (10^7.25^/0.1 ml) was measured using the Reed-Muench method. When observed by electron microscopy, the virus displayed a circular shape, and the surface projections were petal-shaped, with a diameter ranging from 100 to 150 nm, which is similar in size to known TGEVs (Figure 1D).

### Complete genome sequence of the TGEV-HX strain

The full-length genome sequence of the TGEV-HX strain was deduced by combining the sequences of 10 overlapping cDNA fragments. The genome sequence of the TGEV-HX strain was 28,580 nucleotides (nt) long, including the poly A tail. The 5′ portion of the genome contained a 314-nt non-translated region (NTR), and ORF1a (315–12,368) and ORF1b (12,326–20,368), encoding the viral RNA-dependent RNA replicase. The structural proteins S, E, M, and N were found to be encoded by ORFs S (nt 20,365–24,708), E (nt 25,857–26,105), M (nt 26,116–26,904), and N (nt 26,917–28,065), respectively. The three non-structural protein-coding genes were ORF3a (nt 24,827–25,042), ORF3b (nt 25,136–25,870), and ORF7 (nt 28,071–28,307). The 5′ NTR included a potential short AUG-initiated ORF (nt 114–121), beginning within a Kozak sequences (5′-UCUAUGAA-3′). The 3′ end of the genome contained a 273-nt untranslated sequence and a poly (A) tail. Upstream from the poly (A) tail, there was a 5′-GGAAGAGC-3′ octameric sequence .

### The non-structural genes

The replicase genes were composed of ORF1a and ORF1b, which contained a 43-nt common region (nt 12,326–12,368) and a “slippery site” (5′-UUUAAAC-3′, nt 12,333–12,339). The ORF1a gene of TGEV-HX was predicted to encode a protein of 4,017 amino acids (aa), while ORF1b was predicted to encode a 2,680-aa protein. Nucleotide sequence analysis indicated that there were no deletions or insertions in the ORF1ab region of the Miller 6 and Purdue TGEV strains.

ORF3a and ORF3b of TGEV-HX were predicted to encode 72-aa and 244-aa proteins, respectively. No deletions or insertions were found in the ORF3a or ORF3b genes of TGEV-HX. The ORF7 gene of TGEV-HX was predicted to encode a 78-aa protein, which contained the common PP1c-binding motif 5′-RVIFLVI-3′ [14]. No deletions or insertions were found in ORF7 of TGEV-HX.

### The structural genes

**Figure 2:**
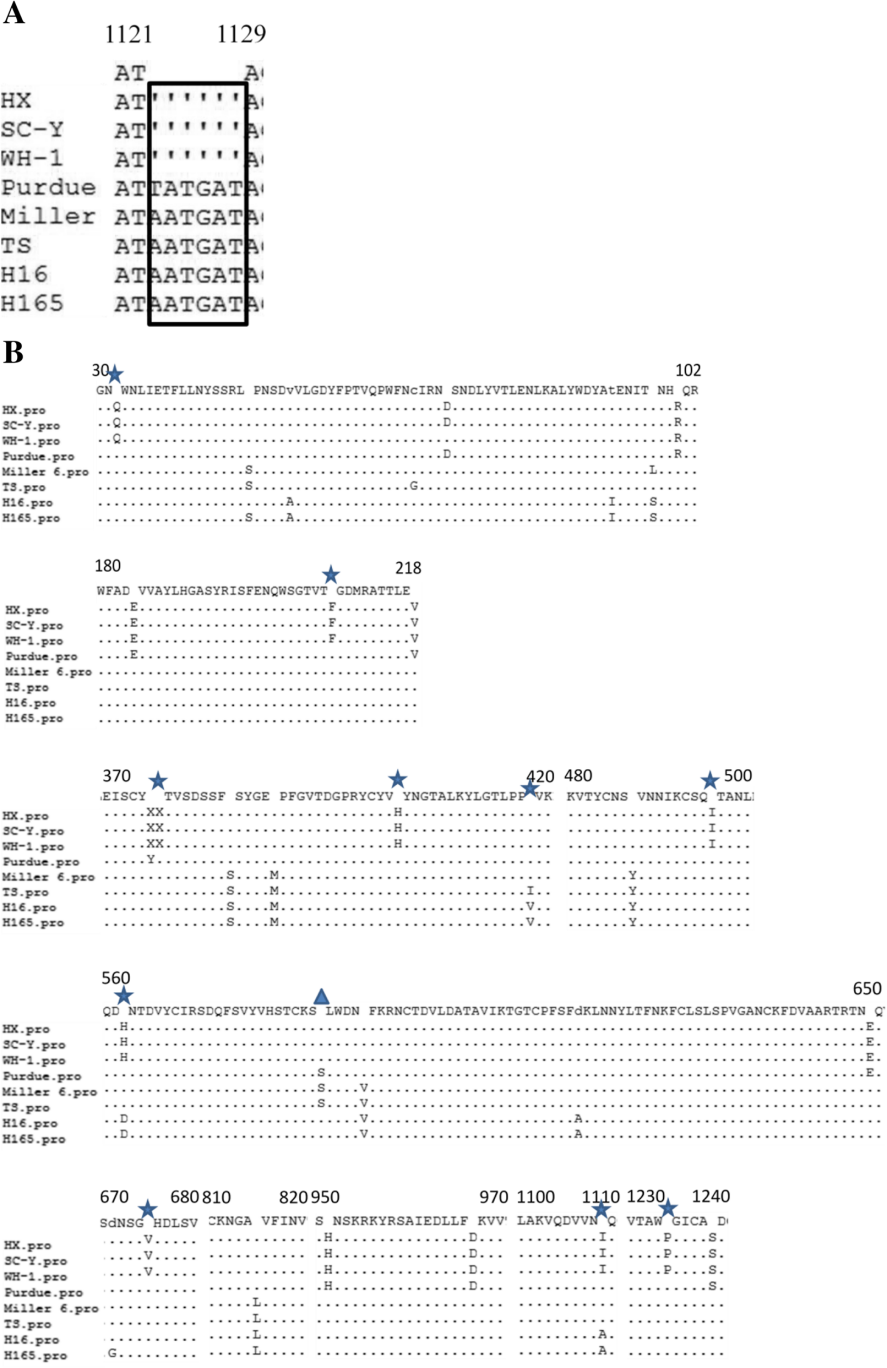
**Multiple sequence alignment of S among TGEV strains. (A)** A 6-nt deletion in the S gene at nt position 1123–1128 of the HX, SC-Y, and WH-1 strains. **(B)** Amino acid alignments of deduced S sequences compared with strain HX. (▲) indicates amino acid 585, (★) indicates amino acids of the HX, SC-Y, and WH-1 strains that are different from those of other transmissible gastroenteritis viruses.

The nucleotide sequence of the S gene of TGEV-HX was 4,344 nt in length, encoding a predicted protein of 1,447 aa. A 6-nt deletion was found in the S gene at nt 1,123–1,128 of the TGEV-HX, SC-Y, and WH-1 strains, which caused the S protein to be two amino acids shorter than those of the Purdue, Miller 6, TS, H16 and H165 strains (Figure 2A). Amino acid 585 of the Purdue, Miller 6, and TS strains was S, while in the TGEV-HX, SC-Y, WH-1, H16, and H165 strains, it was A (Figure 2B). Amino acids 32, 208, 376, 403, 418, 496, 562, 675, 1,109, and 1,234 of TGEV-HX were identical to those of strains SC-Y and WH-1, but different from those of strains TS, H16, and H165.

Sequence analysis revealed no deletions or insertions in the E and N genes of any of the TGEVs. The predicted E, M, and N proteins were 82-, 264-, and 382-aa long, respectively (Table 3).

**Table 3:** **Length, in nucleotide acids and amino acids, of the predicted structural and nonstructural proteins of the transmissible gastroenteritis virus (TGEV)-HX strain**

**HX**	**Position**	**nt**	**aa**
Replicase 1a	315-12368	12054	4017
Replicase 1b	12326-20368	8043	2680
S	20365-24708	4344	1447
ORF3a	24827-25042	216	72
ORF3b	25136-25870	735	244
E	25857-26105	249	82
M	26116-26904	789	264
N	26917-28065	1149	382
ORF7	28071-28307	237	78

### Homology comparison

To investigate the homology of TGEV-HX to other TGEVs, the nucleotide and predicted amino acid sequences of the nonstructural and structural protein coding genes were compared (Table 4). The results suggested that TGEV-HX showed higher identity to strains SC-Y, WH-1, and Purdue, and less identity to TS, Miller 6, and H165.

**Table 4:** **Nucleotide and amino acid sequence identities (%) of various transmissible gastroenteritis virus (TGEV) strains**

	**SC-Y**	**WH-1**	**Purdue**	**Purdue 115**	**H16**	**H165**	**M6**	**M60**	**TS**
ORF1A	99.4/99.1	99.9/99.9	99.8/99.6	99.8/99.8	98.9/98.8	98.9/98.8	99.0/99.0	98.9/98.9	98.8/98.6
ORF1B	99.8/99.8	100.0/100.0	99.9/100.0	99.9/100.0	99.0/99.6	99.0/99.6	99.0/99.6	99.0/99.6	98.9/99.6
S	99.7/99.4	100.0/99.9	99.5/99.0	99.9/99.8	98.1/97.7	98.0/97.6	98.3/98.0	98.2/97.8	98.3/98.0
ORF3A	100.0/100.0	100.0/100.0	99.5/98.6	100.0/100.0	93.1/88.9	92.6/87.5	93.1/88.9	92.6/87.5	92.1/86.1
ORF3B	99.9/99.6	99.9/99.6	99.7/99.2	99.9/99.6	98.9/97.6	98.8/97.1	98.9/97.6	57.4/44.1	98.5/96.3
E	100.0/100.0	100.0/100.0	99.6/98.8	100.0/100.0	98.0/95.2	97.2/92.8	98.4/95.2	98.4/95.2	98.8/96.4
M	99.7/99.2	99.9/99.6	99.7/99.2	99.9/99.6	98.1/97.3	98.1/97.3	98.2/97.7	98.2/97.7	98.0/97.0
N	99.9/99.7	100.0/100.0	99.7/99.7	99.9/99.7	98.2/98.4	98.1/98.4	98.2/98.4	98.0/98.2	98.1/98.2
ORF7	100.0/100.0	100.0/100.0	100.0/100.0	100.0/100.0	96.2/93.7	96.2/93.7	95.8/93.7	95.8/92.4	96.2/93.7

### Phylogenetic analysis

**Figure 3:**
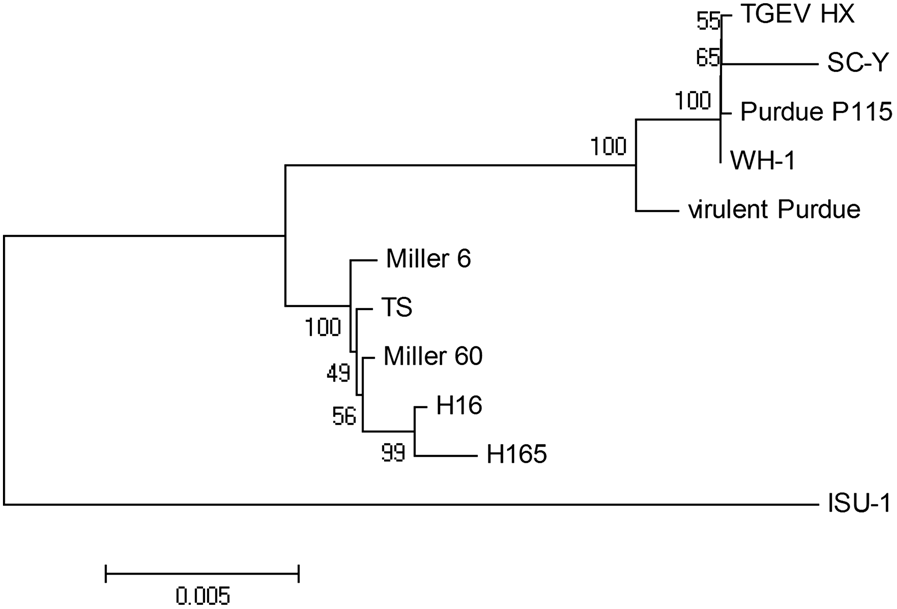
**Phylogenetic tree based on the complete S nucleotide sequence.** The tree was constructed based on the neighbor-joining (NJ) method using MEGA 4.0 software. Bootstrap values were calculated with 1,000 replicates of the alignment.

Phylogenetic analysis of the complete S gene showed that TGEV strains could be divided into two groups (Figure 3). The TGEV-HX strain had a close relationship with the Purdue strain and is more distant evolutionarily from the Miller strains group and strain ISU-1.

## Discussion

As an enteropathogenic coronavirus, TGEV is the major cause of viral enteritis and diarrhea in neonatal pigs, resulting in significant economic losses. Currently, TGEV occurs sporadically in parts of Europe, North America, and Asia. The fact that wild and domestic carnivores (foxes, dogs, cats, and possibly minks) seroconvert to TGEV indicates that they are potential subclinical carriers of TGEV. [15]. In summation, TGEV has become a new challenge for the pig farming industry. As few TGEV genome sequences have been published, little is known about TGEV evolution. The results of this study will provide necessary information for further understanding the evolution of TGEV.

A complete sequence analysis indicated that no deletions or insertions were found in the 5′- and 3′- NTR regions of TGEV-HX, suggesting that its replication and transcription mechanism was not changed. A 6-nt (nt 1,123–1,128) deletion in the S gene was found in the TGEV-HX, SC-Y, and WH-1 strains, but not in the Purdue, Miller 6, TS, H16, and H165 strains. It was showed that this deletion was observed in the attenuated Purdue strains PUR46-C8 and PUR46-MAD, and it was considered to play a role in viral attenuation [16]. Competition studies using monoclonal antibodies led to the prediction of at least four main antigenic sites, designated A, B, C, and D [17,18]. The A and B sites (aa 506–706) have been mapped, and they serve as the major antigenic sites, including the binding site for the viruses host receptor, aminopeptidase N (APN). Single amino acid changes in the S protein can greatly impact its antigenicity [19]. The serine to alanine mutation at amino acid 585 may have a significant influence in receptor binding or neutralizing antibody interactions [18]. Five Chinese strains, TGEV-HX, SC-Y, WH-1, H16, and H165, ha and alanine residue at this position, while the Purdue, Miller 6, and TS strains had a serine residue, which suggested that the antigenicity of the S protein of the five Chinese strains may be changed. Furthermore, amino acids 32, 208, 376, 403, 418, 496, 562, 675, 1,109, and 1,234 of TGEV-HX were identical to those of strains SC-Y and WH-1, but different from those of strains TS, H16, and H165.

The nucleotide and amino acid sequence homology analysis of the structural proteins and non-structural proteins indicated that TGEV-HX was highly similar to the WH-1, SC-Y, and Purdue strains, and had a lower sequence similarity to the Miller 6, TS, H16, and H165 strains. The phylogenetic analysis showed that TGEV-HX was closely related to the SC-Y, WH-1, and Purdue strains, which belonged to the Purdue cluster, while the Miller 6, TS, H16, and H165 strains belonged to the Miller cluster, which was consistent with the results of the homology comparison. The data obtained in this study indicated that HX had different ancestors than the early Chinese strain H16, and it might be derived from the same ancestor as the SC-Y, WH-1, and Purdue strains.

## Conclusions

The present study provides the complete genome sequence of a TGEV-HX strain from China. By comparing the S gene and protein with those of other TGEV strains, we have gained a further understanding of the genetic structure, diversity, and evolution of the TGEV-HX strain. Our next work is to evaluate the characteristics of mutations in the S gene using a reverse genetic approach in animal experiments.

## Abbreviations

TGEV: Transmissible gastroenteritis virus
CoV: Coronaviridae
ORF: Open reading frames
S: Spike
HP: Hydrophobic protein
